# Social-ecological change in the Omo-Turkana basin: A synthesis of current developments

**DOI:** 10.1007/s13280-018-1139-3

**Published:** 2019-01-08

**Authors:** Jennifer Hodbod, Edward G. J. Stevenson, Gregory Akall, Thomas Akuja, Ikal Angelei, Elias Alemu Bedasso, Lucie Buffavand, Samuel Derbyshire, Immo Eulenberger, Natasha Gownaris, Benedikt Kamski, Abdikadir Kurewa, Michael Lokuruka, Mercy Fekadu Mulugeta, Doris Okenwa, Cory Rodgers, Emma Tebbs

**Affiliations:** 1 Department of Community Sustainability, Natural Resources Building, 480 Wilson Road, East Lansing, MI 48824 USA; 2 Department of Anthropology, Durham University, South Road, Durham, DH1 3LE UK; 3 Department of Geography, University of Cambridge, Downing Place, Cambridge, CB2 3EN UK; 4 School of Agriculture and Veterinary Sciences, South Eastern Kenya University (SEKU), P.O. Box 170-90200, Kitui, Kenya; 5 School of Geography, Geology and the Environment, University of Leicester, Bennett Building, University Road, Leicester, LE1 7RH UK; 6 Department of Anthropology, College of Social Sciences and Humanities, Hawassa University, Hawassa, Ethiopia; 7 Laboratoire d’Anthropologie Sociale, 3 Rue d’Ulm, 75005 Paris, France; 8 St John’s College, University of Oxford, Oxford, OX1 3JP UK; 9 Department Integration & Conflict, Max Planck Institute for Social Anthropology, Advokatenweg 36, 06114 Halle/Saale, Germany; 10 4247 Palatine Avenue North, Seattle, WA 98103 USA; 11 Arnold Bergstraesser Institute, Windausstr. 16, 79110 Freiburg I Br, Germany; 12 University of York, Heslington, York, Yorkshire YO10 5DD UK; 13 Department of Food Science and Nutrition, School Of Agriculture and Biotechnology, Karatina University, P.O. Box 1957-10101, Karatina, Kenya; 14 Institute for Peace and Security Studies, Addis Ababa University, PO BOX 1176, Addis Ababa, Ethiopia; 15 Department of Anthropology, London School of Economics and Political Science, Houghton Street, London, WC2A 2AE UK; 16 School of Anthropology and Museum Ethnography, Institute of Social and Cultural Anthropology, 51 Banbury Road, Oxford, OX2 6PE UK; 17 Department of Geography, King’s College London, Bush House (NE) 4.01, 40 Aldwych, London, WC2B 4BG UK

**Keywords:** Ecosystem services, Equity, Gibe III, Omo, Social-ecological systems, Turkana

## Abstract

This paper synthesizes current knowledge on the impacts of the Gibe III dam and associated large-scale commercial farming in the Omo-Turkana Basin, based on an expert elicitation coupled with a scoping review and the collective knowledge of an multidisciplinary network of researchers with active data-collection programs in the Basin. We use social-ecological systems and political ecology frameworks to assess the impacts of these interventions on hydrology and ecosystem services in the Basin, and cascading effects on livelihoods, patterns of migration, and conflict dynamics for the people of the region. A landscape-scale transformation is occurring in which commodities, rather than staple foods for local consumption, are becoming the main output of the region. Mitigation measures initiated by the Ethiopian government—notably resettlement schemes—are not adequately buffering affected communities from food insecurity following disruption to indigenous livelihood systems. Therefore, while benefits are accruing to labor migrants, the costs of development are currently borne primarily by the agro–pastoralist indigenous people of the region. We consider measures that might maximize benefits from the changes underway and mitigate their negative outcomes, such as controlled floods, irrigating fodder crops, food aid, and benefit sharing.

**Electronic supplementary material:**

The online version of this article (10.1007/s13280-018-1139-3) contains supplementary material, which is available to authorized users.

## Introduction

Semi-arid regions across Africa are undergoing a period of rapid environmental and social change, but the Omo-Turkana region in southern Ethiopia and northern Kenya is arguably unique in the scale and pace at which change is occurring. This paper focuses on the changes taking place due to a series of current and planned future hydropower dams in the Lower Omo, the most recently completed being the Gilgel-Gibe III (hereafter ‘Gibe III’) dam, Africa’s tallest, and the establishment of irrigated sugar estates covering a projected 100 000 hectares (ha) as well as large-scale cotton schemes (Sugar Corporation 2013). Since 2015, the regulation of river flow by the dam has eliminated the annual flood pulse of the river, and the filling of the Gibe III reservoir has reduced the water level of Lake Turkana (USDA Foreign Agricultural Service 2017), with abstraction of water for irrigation expected to cause further declines in lake levels. These changes are of geopolitical significance: although the whole course of the Omo River is contained within Ethiopia, the river terminates in Lake Turkana, which lies entirely within Kenya. While the threat that current developments in the region pose to indigenous livelihoods has received attention from advocacy groups (e.g., Human Rights Watch, Oakland Institute, Survival International, International Rivers) and indigenous NGOs (e.g., Friends of Lake Turkana, Sarima Indigenous Peoples’ Land Forum) there has so far been relatively little attention from the scientific community. The most comprehensive existing analyses suggest that the vast majority of the Basin’s population stands to be negatively affected by ongoing hydrological and land-use changes (Avery 2010, 2012a, b; Carr 2016).

### Conceptual framing

The study of social-ecological systems (SES) provides an analytical framework through which system dynamics can be identified and therefore constitutes a suitable framing for this topic (Walker et al. 2004). Given the landscape-wide impact of developments in the Basin, data are required that represent all elements of the SES, and thus we present a multidisciplinary study focused around a system driver (the technological and engineering developments) in a bounded geographical location, the Omo-Turkana Basin. This paper synthesizes data regarding current developments on both sides of the political boundary between Ethiopia and Kenya and integrates social, political and ecological perspectives on the cascading changes resulting from engineering interventions upstream, and their implications for indigenous livelihoods. Since its emergence, social-ecological systems theory has integrated the concept of ecosystem services (ESS) (the services humans receive from nature (TEEB 2010)) as a way of understanding the links from the ecological subsystem to the social subsystem (Holling 1986; Biggs et al. 2015). Critiques of SES theory from the social sciences relate to under-theorization related to resource conflicts and the importance of power asymmetries, i.e., the dynamics within the social subsystem that in turn influence the ecological subsystem. Therefore in this paper we integrate a political ecology framework, which presupposes that changes in the environment do not affect populations in a homogenous way, considering “whose needs are being met from the goods and services” (Beymer-Farris et al. 2012, p. 283) and conversely which groups are accruing the most significant costs (O’Brien and Leichenko 2003). This allows us to create a conceptual model of the Omo-Turkana Basin SES disaggregated into different populations, and to explore how changes in the SES affect these populations differently.

### Study site

**Fig. 1:**
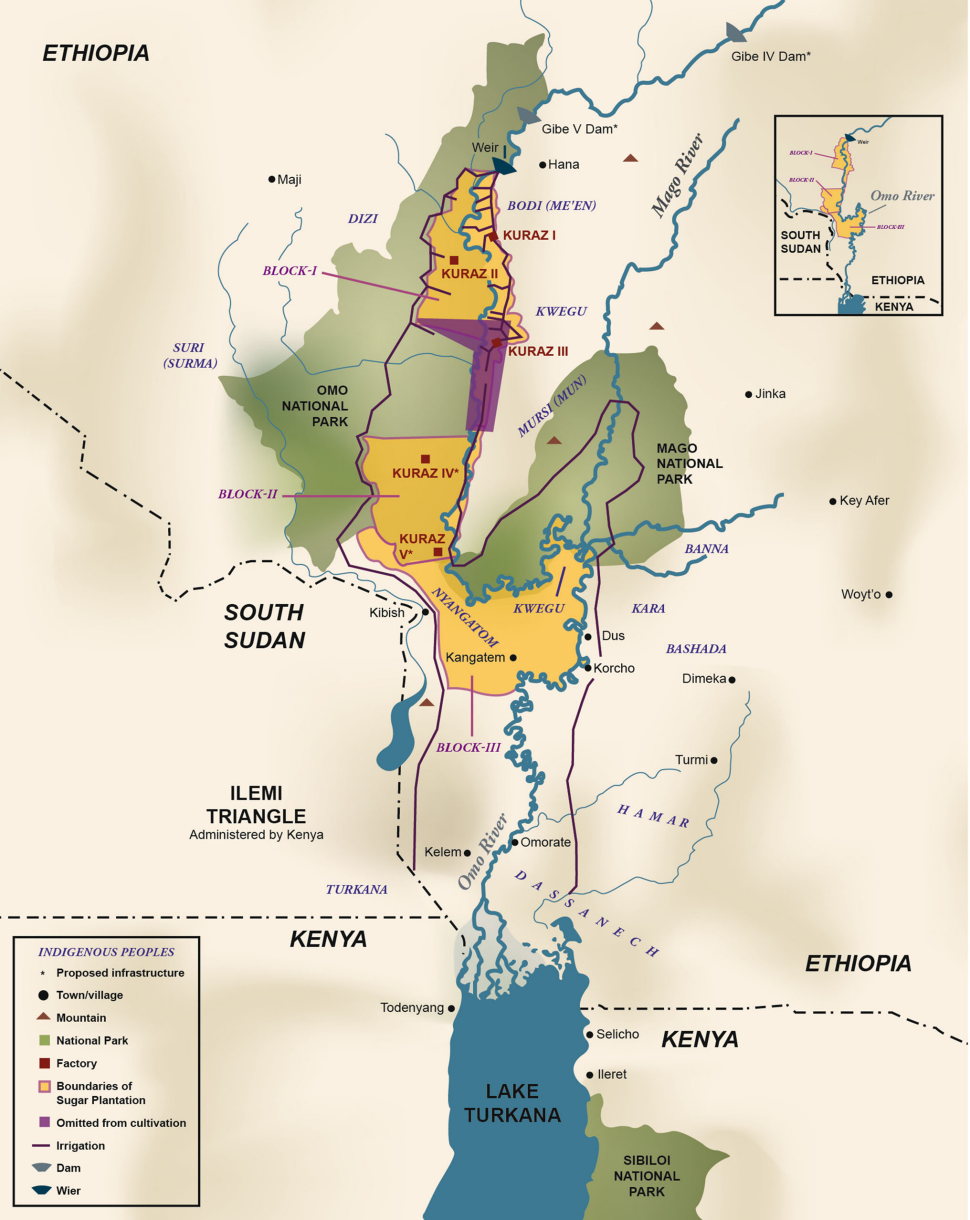
The boundaries of the social-ecological system under study—the Lower Omo Valley in Ethiopia, and Lake Turkana in Kenya, of which the northern part is shown here

The hydrological boundaries of the whole Turkana Basin create an area of approximately 131 000 km^2^ in the Horn of Africa (Feibel 2011). This paper focuses on developments in a section of the basin, the Lower Omo Valley, and the impact of these developments on downstream communities and ecosystems, including Lake Turkana and its surrounding lands, as shown in Fig. 1, and henceforth referred to as the Omo-Turkana Basin.

The Omo River supplies 90% of the inflow to Lake Turkana, the world’s largest permanent desert lake and the world’s largest alkaline lake, with the remaining water balance coming from the Turkwell River (Butzer 1970; Hopson 1982; Avery 2010). The Basin is hydrologically closed; hence, the only output of water is through evaporation (Avery 2010). The population that stands to be affected by the technological developments belong mostly to indigenous groups practicing diverse livelihood strategies including pastoralism, agro-pastoralism, and fishing (Carr 2012, 2016). They number approximately 1 000 000 people (Table 1).

**Table 1:** Populations that stand to be most affected by developments in the Lower Omo Valley and Lake Turkana environs, based on most recent census data for relevant districts (Ethiopia) and counties (Kenya) (Central Statistical Agency 2008, 2011; Kenya National Bureau of Statistics 2009). Given immigration into the region and high growth rates, these figures are conservative

	District (Ethiopia) or County (Kenya)	Area (sq km)	Latest census population
Ethiopia	Selamago	4450	27 866
	Nyàngatom	2649	17 640
	Dásanach	2102	52 708
Kenya	Turkana	71 598	855 399
Total		80 799	953 613

## Materials and methods

This paper is a product of a multistage protocol that integrates expert elicitation and a scoping review. When addressing decision making regarding natural resources, empirical data are often not available from which to develop the predictions of management decisions, thus it is common to reply on expert judgment to develop estimates for outcomes (Runge et al. 2011). As Runge et al. (2011, p. 1216) summarize—“formal expert elicitation is a means towards a structured way to address such uncertainties. It refers to a structured approach of consulting experts on a subject where there is insufficient knowledge and seeks to make explicit the published and unpublished knowledge and wisdom of experts.” Given the data gaps in the published literature regarding recent change in the Omo-Turkana Basin, we initiated activities to bring together experts in the region from across different disciplines. The majority of expert elicitations are used to quantify ranges for poorly known parameters (i.e., using the Delphi process and its variants), but they may also be used to further develop qualitative issues such as definitions, assumptions or conceptual models (Knol et al. 2010). Given the lack of landscape-scale studies of the Omo-Turkana Basin and thus uncertainty around which parameters to include, we took the latter route—bringing together experts to create a conceptual model of the landscape-scale SES, thus creating the framework for a scoping review to be carried out collectively by the same group of experts. We followed the seven-step process outlined by Knol et al. (2010) for expert elicitation: (1) characterization of uncertainties,—i.e., what are the elements of the Omo-Turkana Basin SES? (2) scope and format of the elicitation—what are the impacts of the technological developments on these elements in the SES? (3) selection of experts; (4) design of the elicitation protocol; (5) preparation of the elicitation session; (6) elicitation of expert judgments; and (7) possible aggregation and reporting.

Step 3 was a prolonged process culminating in July 2016, when Hodbod and Stevenson hosted a workshop in Nairobi attended by 17 researchers (henceforth referred to as experts). During the 18 months prior to the workshop, Hodbod and Stevenson used a snowball sampling (Bryman 2015) process to initiate a multidisciplinary network of approximately 40 researchers who have active research projects in the region. The network is an international consortium of social and environmental scientists researching the impacts of hydrological, agricultural, and economic change on the societies and ecosystems of the Omo-Turkana Basin, the Omo-Turkana Research Network (OTuRN, https://www.canr.msu.edu/oturn/). Workshop participants were selected from the network by Hodbod and Stevenson to ensure representation across disciplines (in natural and social sciences and humanities) and of the presumed critical elements of the social-ecological system, i.e., hydrology, water quality, ecosystem services, wildlife ecology, food security, political science, and livelihoods. The participants were 65% male and 35% female; represented multiple levels of seniority, from professors to PhD candidates; and were from academic institutions in five countries [Kenya (5), UK (5), Germany (4), Ethiopia (2), and USA (1)]. The rapid pace of developments in the region means that graduate students make up a substantial proportion of those currently carrying out relevant field studies (~ 40%).

**Fig. 2:**
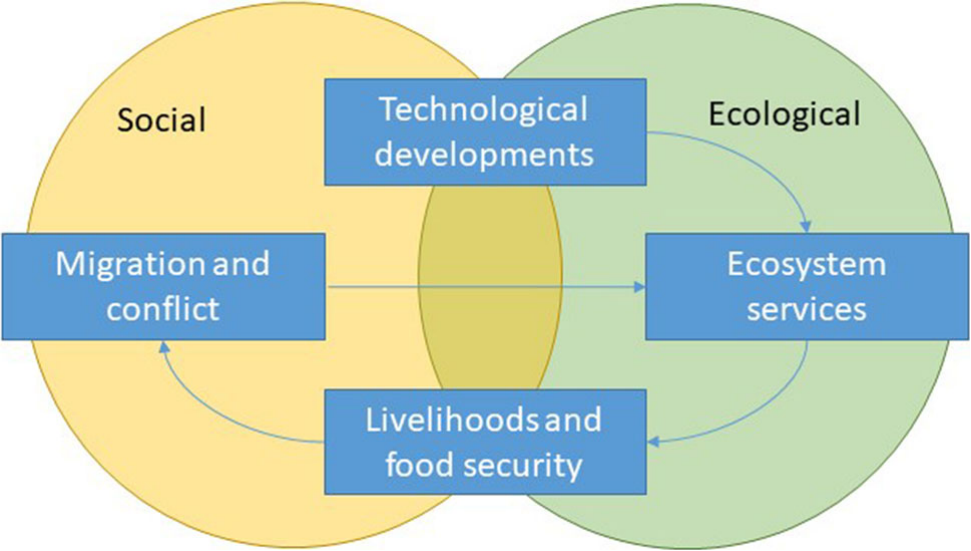
Conceptual model of the key elements within the Omo-Turkana Basin SES. Technological developments refers to dam construction and large-scale irrigated agriculture. Elements affected within the ecological subsystem include flood regime, lake levels, biodiversity, and irrigation potential

Steps 4 and 5 followed an expert elicitation process based on the qualitative, in-person elements of the expert elicitation process (Ayyub 2001; Swor 2011) and involved an iterative process of mapping the elements of the SES in order to create a final model achieved by consensus and presented in Fig. 2.

Steps 6 and 7 began in Nairobi, where experts presented their own findings on how the technological developments were creating change in the specific element of the SES their research studied. The elements on which we focus in this paper fulfill the following criteria: (a) change has already begun to occur, (b) we have data to begin to analyze, and (c) these changes pose the most severe potential impacts on livelihoods. Appendix S1 outlines the disciplinary expertise of the workshop participants and authors, and the datasets used by each to inform the expert elicitation and scoping review. There are elements of the SES where data do not exist—these are highlighted in the conclusion. Given that Gibe III is the most significant major technological development in the region, the majority of data relate to this intervention.

Each participant followed a scoping review methodology to create a written summary of SES impacts, based on both published and unpublished data. Given the rapid nature of changes in the region, and the early stage of career of those who have carried out timely and relevant fieldwork (~40% of our participants being graduate students), a considerable proportion of the data are unpublished. Scoping reviews aim to map the existing literature in a field to examine the extent and nature of research activity in that area; to determine the value and potential scope of undertaking a full systematic review; to summarize and disseminate research findings; and to identify research gaps in the existing literature (Arksey and O’Malley 2005; Levac et al. 2010; Pham et al. 2014). While we were not planning the review to be a precursor to a systematic review, we aimed to achieve the other three goals: to demonstrate the extent of existing research, to share research findings, and to expose gaps in the literature. Once written, the individual elicitations were compiled by Hodbod and Stevenson and then the paper draft was sent for iterative editing by the authors until there was consensus on the presentation of data and the equity analysis presented within the discussion. The results section is organized as shown in Fig. 2. After describing the intervention and study context, we present data on impacts on the ecosystem, and impacts on social systems. The final sections summarize these findings in light of a social-ecological systems framing, and appraise the distribution of costs and benefits among stakeholders.

## Context of technological developments

The lowlands of the Omo Valley and the area surrounding Lake Turkana are home to people whose livelihood systems are based on a combination of mobile pastoralism, flood-retreat and rain-fed farming, and fishing—a portfolio of economic strategies that allow them to exploit seasonally fluctuating resources (Turton 1988; Leslie and Little 1999). The semi-arid climate of the Lower Omo and the scarcity of readily extractable resources made this region one of the last to be incorporated into the expanding Ethiopian and British empires in the late nineteenth and early twentieth centuries (Lamphear 1992; Markakis 2011). Until the 1970s, Ethiopian state representation in the Lower Omo was limited (Tornay 1993; Almagor 2002). Although state administration (e.g., border policing and provision of famine relief) was more extensive on the Turkana side under the British colonial administration, development activities on both sides of the border remained minimal until the 1960s, when programs of sedentarization and the establishment of the Omo National Park (1966) and Mago National park (1979) led to increased resource pressures for many of the region’s inhabitants (Turton 2011).

Planning and construction of the Gibe III dam began in 2005, but the initiative has precedents in the development plans drawn up by Ethiopia’s military regime in the 1980s (WRDA 1984) and the Omo Basin Development Plan, financed by the African Development Fund in the 1990s (Woodroofe & Associates and Mascott Ltd. 1996). The Gibe III is the first large dam on the Omo; it takes its name from a tributary of the Omo on which two previous hydroelectric turbines, Gibe I and II, were installed between 2004 and 2010. The catchment area of the dam contains 67% of the total Basin flow, and the 200 km^2^ reservoir will have a storage capacity of 2 240 000 m^3^ (Salini Impregilo 2016). The production of electricity is important to both the Ethiopian and Kenyan states and is one of the outcomes of the project intended to redress inequalities (along with jobs and foreign revenue)—the 246-m-high dam is intended to have an installed capacity of 1 870 MW, representing an 80% increase to the country’s current generation capacity, with half the produced electricity remaining in Ethiopia, 500 MW being exported to Kenya, 200 MW to Sudan, and 200 MW to Djibouti (Avery 2013; Salini Impregilo 2016; Central Intelligence Agency 2017).

In addition to generating electricity, Gibe III’s regulated flows enable large-scale irrigated agriculture in the lower catchment of the Omo. The availability of water for agricultural use, together with favorable investment conditions proposed by the Ethiopian government, has attracted considerable interest from domestic and foreign investors. The largest of the estates currently under development, the Kuraz Sugar Development Project (KSDP), launched by the state-owned Ethiopian Sugar Corporation (ESC), is projected to cover 100 k ha and to serve four new sugar factories (Sugar Corporation 2013; Kamski 2016a). The current extent of the sugar estate is also rivaled in size by the cumulative total of private estates allocated to foreign and domestic investors, which include cotton and palm oil (Kamski 2016b).

## Environmental impacts

The Gibe III dam and commercial plantations will affect both the quality and quantity of water available to downstream regions. Below, we expand on the hydrological, ecological, and biological impacts and address the implications of these changes for people living within the Basin.

### Hydrology

The Gibe III dam has permanently dampened seasonal flow variability in the Omo River, eliminating the annual flood pulse. Assessing the impact of Gibe III has been complicated by the lack of flow data for the Omo River, last measured by the Ethiopian Water Resources Authority in 1980 (Avery 2009). Hence, hydrological studies of the Basin have made use of satellite datasets to model the water balance (Avery 2009, 2012a, b; Velpuri and Senay 2012). Before the construction of the dam, the Omo had a fluctuating flow rate and flooded annually between July and October, and Lake Turkana water levels fluctuated between 1 and 1.5 m each year (Avery 2012a). The Gibe III reservoir began to fill in February 2015 and in October 2015, the reservoir level reached 200 m (the minimum level to begin power generation), 40 m below the dam’s crest (Salini Impregilo 2016). The filling of the Gibe III reservoir (completed in December 2016) caused a decline in Lake Turkana water levels of ~ 1.5 m between January 2015 and January 2017, as shown by satellite radar altimetry data (USDA Foreign Agricultural Service 2017), consistent with predictions of Avery (2009, 2012a, b and Velpuri and Senay (2012). Further reductions in lake levels are expected due to irrigation abstractions, with the magnitude of decline depending on the final extent and management of the new plantations. A worst-case scenario would be the complete drying of Lake Turkana, prompting analogies with the Aral Sea disaster (Avery 2012a, b; Stevenson 2018a).

**Fig. 3:**
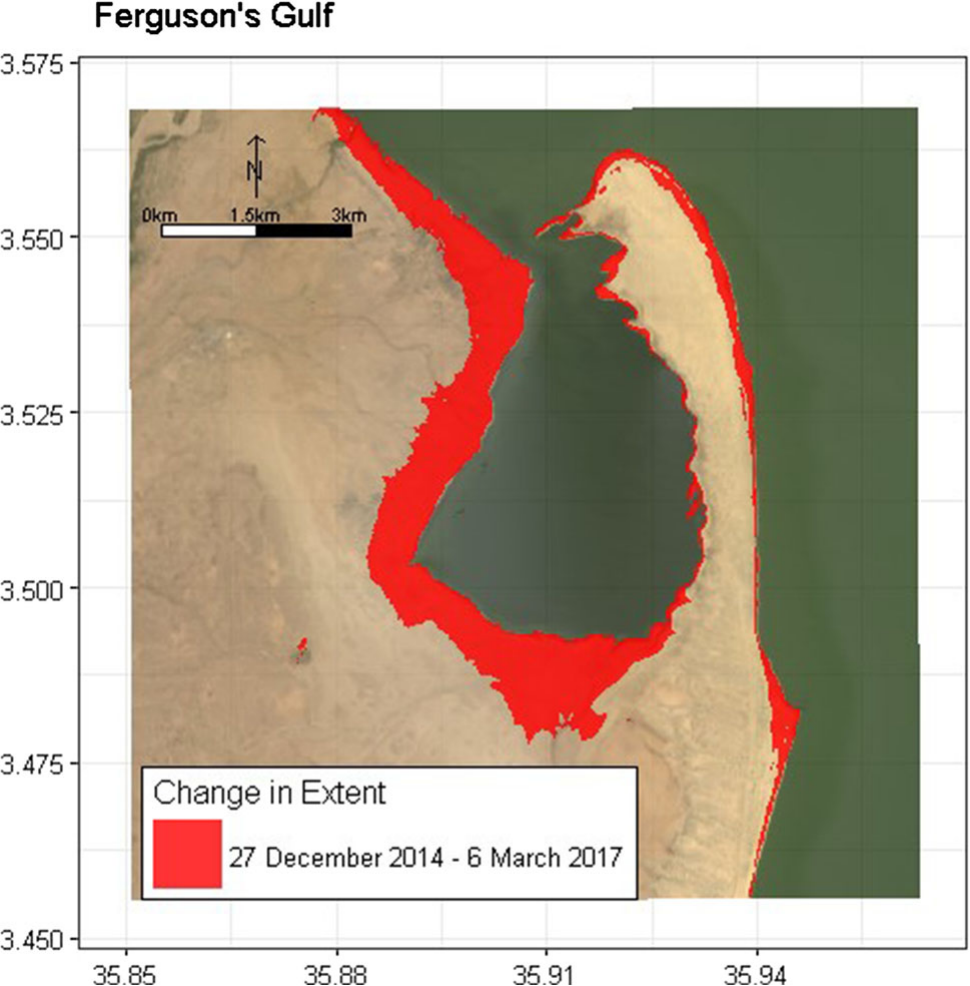
Change in surface water extent around Ferguson’s Gulf, Lake Turkana, between December 27, 2014 and March 6, 2017. Surface water extent was estimated by applying the Modified Normalised Difference Water Index to NASA Landsat 8 surface reflectance imagery downloaded from the USGS Earth Explorer website (https://earthexplorer.usgs.gov/)

As a result of declining lake levels, the shoreline of Ferguson’s Gulf, an important area for fishing and fish breeding, and the only sheltered area on the western side of the lake, has receded significantly, and the mouth of the Gulf has narrowed to less than 1 km (Fig. 3). Further declines in lake levels due to irrigation abstractions would result in a complete drying of Ferguson’s Gulf, leading to a loss of fishing livelihoods for surrounding communities and exacerbating food insecurity in the region. While the Gulf has dried completely during past periods of low lake levels (Kolding 1993; Avery 2010), these previous dry periods were a result of natural climate fluctuations rather than human activities.

### Ecology

Changes in the volume and seasonality of inflows from the Omo will impact significantly on the ecology of downstream regions, including Lake Turkana. The Omo has historically carried high sediment loads and vital nutrients to the Lower Omo and Lake Turkana (Avery 2012a, b). The Gibe III dam traps sediment bedload, but passes suspended and colloidal sediments, which will overall reduce downstream sediment transport. Regulation of flow for hydropower and irrigation also implies eliminating the seasonal flood pulse. These hydrological changes will have impacts on riverine communities dependent on sediments and floodwater for farming (see below, under the section: “Social and Economic Impacts”).

The dampening of seasonal inflows from the Omo are also expected to cause a decline in downstream productivity, given that the nutrient inputs from the river are essential for primary productivity in the lake (Tebbs 2014). While primary productivity levels in Lake Turkana are low compared to some freshwater systems (e.g., Lake Victoria), they are higher than others (e.g., Lake Tanganyika), and the productivity rates in sheltered areas and shallow lagoons such as Ferguson’s Gulf are among the highest ever recorded (up to 2 710 mg m^−3^ Kallqvist et al. 1988). Lake Turkana’s distinctive jade green color is caused by high concentrations of cyanobacteria (blue–green algae) in the lake’s waters, which are key to sustaining the lake’s productive fisheries (Kolding 1993). The lake is nutrient limited (Kallqvist et al. 1988), and input from the Omo is a critical factor driving chlorophyll concentrations, particularly in northern parts of the lake, as waters entering from the Omo River carry vital nutrients into the lake (Hopson 1982; Avery 2013; Gownaris et al. 2016). As a result, there is a strong productivity gradient decreasing from the north to south and seasonal phytoplankton blooms coincide with the annual flooding from the Omo River (Tebbs et al. 2015).

### Biodiversity

Given the breeding grounds for many of Lake Turkana’s fishes are found in areas that are seasonally covered by the fluctuating depth of the lake, the lake’s fish are at risk under the post-dam hydrological regime. Lake Turkana is home to over sixty fish species (Butzer 1970), of which ten are endemic to the lake (Froese and Pauly 2017). Unlike in some other African lakes, Nile tilapia (*Oreochromis niloticus*) and Nile perch (*Lates niloticus)* are native to Lake Turkana and are the highest valued species in the lake’s commercial fishery (Ojwang et al. 2008; Gownaris et al. 2016). The hydrological changes stemming from upstream development will potentially reduce the lake biomass by up to 50% (Avery 2012a, b) and recent studies estimate that it will reduce Lake Turkana’s fisheries’ productivity by more than two-thirds, potentially leading fisheries to collapse (Gownaris et al. 2015, 2016). These projections correspond with historical changes in the lake, as falling water levels during the 1970s to 1980s are thought to have contributed to severe reductions in some fish populations at that time (Kolding 1995; Muška et al. 2012) and to drastic changes in the structure of the lake’s food web (Kolding 1993).

The sensitivity of Lake Turkana’s fishes to upstream development is based primarily on three factors: physiological tolerance to salinity, changes in nutrient availability, and the sensitivity of the fishes’ reproductive ecology. First, reduced inflow from the Omo River will lead to concomitant increases in Lake Turkana’s salinity, given the closed nature of the basin (Avery 2012b). With the exception of tilapia, a group known for its environmental flexibility (e.g., Stickney 2005), the salinity tolerance of the lake’s fishes is unknown. However, the lake’s salinity is already restrictive for fishes, some of which (e.g., mormyrids) are found only in the Omo Delta region, where salinity is lowest. Second, reduced inflow from the Omo River may also lead to a reduction in the lake’s nutrient levels (although, as an indicator of the uncertainties involved, fertilizer use upstream could result in a countervailing increase in nutrient inputs) (Tebbs 2014). Species with high dietary flexibility may be able to cope with food web changes, but some of the lake’s commercially important species (e.g., *Tilapia zillii*) have specialist diets that will limit their resilience (Gownaris et al. 2015). Third, the reproductive ecology of the lakes fishes is a crucial factor. Lake Turkana’s fishes rely on physiochemical changes related to the seasonal flood pulse as a signal to breed (Hopson 1982). These breeding signals are particularly important for potadromous species. Changes in water inflow patterns are also likely to alter breeding habitat availability in the lake and the Omo River (Gownaris et al. 2016).

Plantation development is also anticipated to reduce biodiversity in the Lower Omo. The transition to year-round monocropping on artificially irrigated land inevitably involves a reduction in biodiversity (Hodbod et al. 2016), and the destruction of vegetation along the river in the service of agricultural development has already deprived many species of their habitat, including honeybees, ungulates, and other wildlife and plants (Buffavand 2016).

## Social and economic impacts

The Gibe III dam, the establishment of commercial agriculture, and the accompanying expansion of road and electricity networks go a long way toward integrating the Lower Omo and Turkana into the Ethiopian and Kenyan economies with the presumption this will benefit local populations, but they also pose major challenges to indigenous livelihoods. Formal assessments of the environmental and social impacts (ESIAs) of Gibe III were prepared only after the start of construction works in 2006 (CESI & Mid-Day International Consulting Engineers 2009; Government of Ethiopia & Ethiopian Electric Power Corporation 2009) (see Monteil and Kinahamn (2015) for an overview). In contrast to the opinions of independent experts (e.g., Africa Resources Working Group 2008; Avery 2009, 2013), these official assessments made light of the implications of the dam for downstream populations. Further, none of the published ESIAs addressed the impacts of plantation development for the ecosystems or livelihoods of the Lower Omo and Lake Turkana. The livelihood systems of the indigenous communities of the Lower Omo depend on the annual flood of the Omo and on access to the land along the river. The end of the floods, the expropriation of some of the most fertile lands for commercial agriculture, and the potential collapse of Lake Turkana’s fisheries present major challenges to traditional livelihoods and thus regional food security. Below we review these implications, and consider their influence on migration patterns and conflict dynamics.

### Livelihood and food security outcomes

About 90 000 people comprising at least ten ethno-linguistic groups depend on flood-retreat farming for sorghum cultivation along the Omo (Turton 2010). In the more southerly parts of the Lower Omo, the areas flooded by the river are larger, while the surrounding plains are dryer; thus, these populations are even more reliant on flood-retreat farming than their more northerly neighbors, who also practice rain-fed cultivation in the plains. All of the indigenous communities of the region depend on livestock for their livelihoods (cattle, sheep, goats) but to different degrees—e.g., the Mursi, with relatively large herds, versus the Kwegu, who are reliant to a greater extent on fishing. Within communities, extended families allocate labor to a diversity of livelihood strategies, including pastoralism, rainfed farming, and flood-retreat farming, in order to maximize access to both animals and crops. For example, the Bodi divide family labor between herding camps and cultivation sites, with camps making seasonal moves that may span a radius of 6 km per year (Fukui 2001). The river’s flood has also been important for livestock herding in the delta: it regenerates some of the most productive grazing land (Carr 2012). The riverine forests of the lower reaches of the river and delta also constitute a valuable resource for foraging and hunting. For most of the region’s indigenous peoples, wildlife and wild plants (e.g., large ungulates, wild greens) are important supplements to the staple diets of sorghum, maize, and dairy products, while honey production is an additional source of income (Buffavand 2016).

Since the end of the floods in 2015, most populations in the northern part of the Lower Omo are entirely dependent either on rain-fed cultivation for crop production, making them highly vulnerable to drought, or (for those allocated irrigated plots within the KSDP) on corporation-controlled irrigation systems (Stevenson and Buffavand 2018). Reductions in the river flows downstream of the plantations are anticipated to reduce the availability and diversity of forage and thereby compromise livestock herding opportunities (Carr 2012). Land clearance for sugar plantations has deprived wildlife of vital habitat and narrowed the portfolio of subsistence opportunities open to the people of the region (Buffavand 2016).

Few scholars have studied the food security of other groups within the region, but household food security surveys conducted in 2013 among Bodi people both showed high levels of food insecurity among both groups, discussed further below (Stevenson and Buffavand 2018). Site visits in the Lower Omo between 2016 and 2018 and ongoing communication between the authors and affected communities provide multiple lines of evidence for increased food insecurity, exacerbated by the drought affecting southern Ethiopia and northern Kenya (Fekadu et al. 2018). So far, social and economic impacts immediately around Lake Turkana are less pronounced than those in the Lower Omo, but the threat to the lake’s fisheries has serious implications for the food security of communities in Turkana (Avery 2013; Gownaris et al. 2015). Fish stocks from Lake Turkana directly benefit local populations of c.200 000 Turkana, Dasanach, and El Molo, and are sold all over Kenya as well as in Uganda and the eastern D.R. Congo (B. Kamski, personal communication, August 26, 2018). Communications from the region suggest that local fishers are already experiencing reduced fish catch (N. Gownaris, personal communication, October 1, 2016). Reduced water and fodder availability would also impact herders in Kenya (Carr 2017).

### Migration implications

A frequently observed response to food insecurity and changes in availability of natural resources is increased mobility, as communities seek compensation in “buffer zones” or in the territory of neighboring groups (Turton 1985, 1988). In the Lower Omo, movements by agro-pastoralists in search of grazing and farmland are currently occurring alongside three other kinds of migration: villagization programs that are aimed at sedentarizing the region’s pastoralist groups; government-planned resettlement of people from more densely inhabited regions of southern Ethiopia; and an influx of migrants from Ethiopia’s southern highlands in search of job opportunities.

**Fig. 4:**
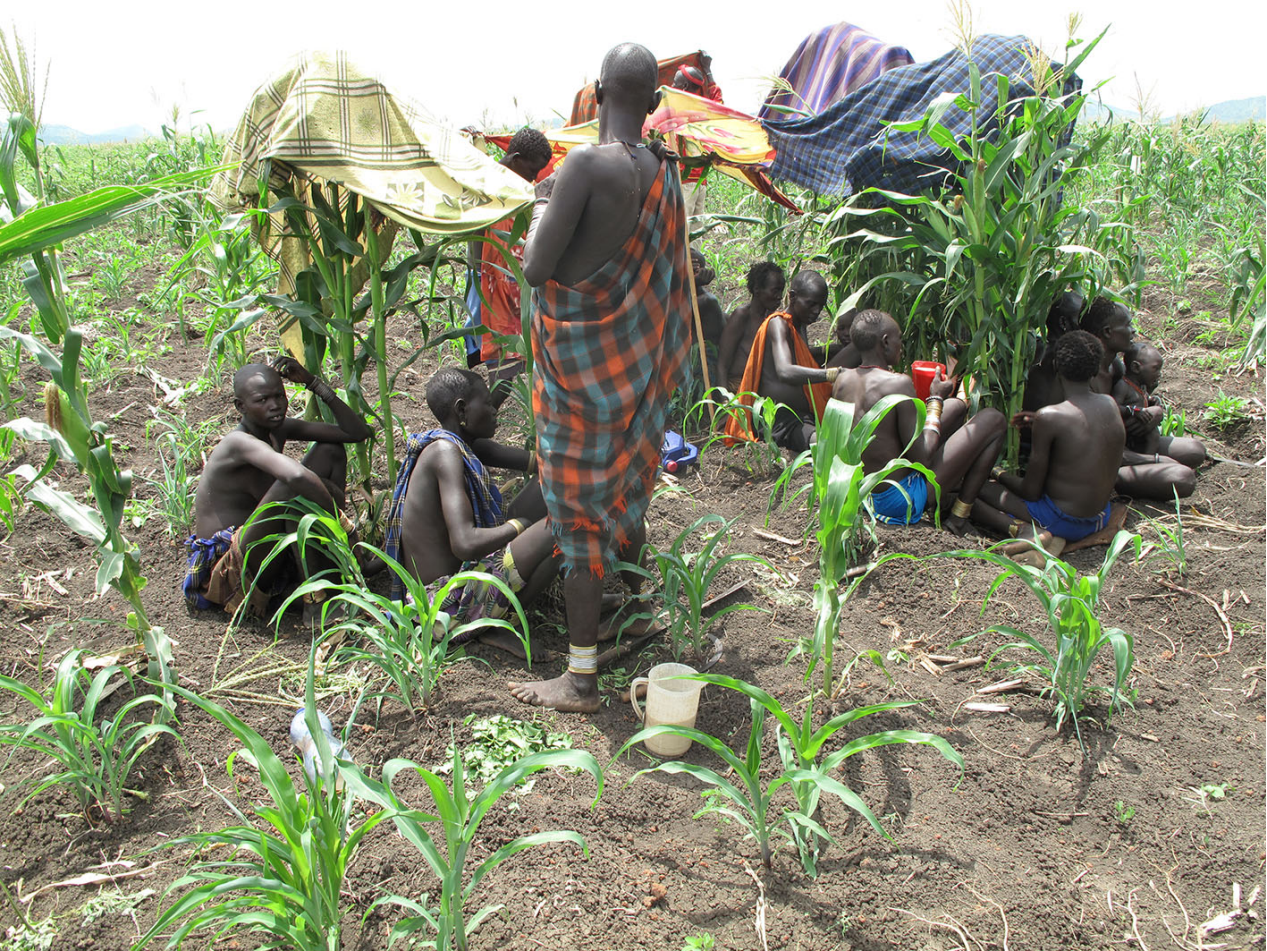
During a break from weeding, rigging up of a makeshift shelter by workers for drinking local beer. Irrigated plot for maize cultivation, Salamago Wereda, Lower Omo. (Photograph courtesy of Lucie Buffavand)

Although the government originally envisioned resettling the entire agro-pastoral population of the South Omo zone in conjunction with the expansion of commercial farms (FDRE 2012), it has so far attempted to implement villagization only in the vicinity of the Kuraz Sugar Development Project, in Salamago Wereda (Block 1 on Fig. 1) and in Hamar (Yidneckachew 2015). Following a campaign in which locals were informed of the villagization plan, settlers were induced to move into new villages in Salamago in 2012 (Yidneckachew 2015). Communities subject to villagization were allotted irrigated plots of 0.25–0.5 ha for production of maize (Fig. 4), but the households who attempted to farm these plots struggled to feed themselves and remained largely dependent on food aid (Stevenson and Buffavand 2018). Conflicts with members of other groups resettled in Salamago, (notably those from Konso) and the withdrawal of food aid, subsequently led to the dissolution of villagization sites (Stevenson 2018b). In other parts of the region where villagization has been attempted, e.g., in Hamar, it has met with strong resistance (Lydall 2015).

In 2004, a fertile area in Bodi land was chosen to resettle about 5 000 people form Konso, in the southern highlands (Ayke 2005). The arrival of settlers from Konso has increased population pressure and competition for farming and grazing land for the Bodi, and have effectively closed off access to some of their most valuable territory for rain-fed farming (Buffavand 2016; Stevenson and Buffavand 2018). These pressures have been intensified by the influx of labor migrants in search of employment on construction projects and the sugar estates (Stevenson and Buffavand 2018).

### Conflict implications

Population movements often lead to conflict (Abbink 2009; Carr 2012). While conflict among the peoples of the Lower Omo and Lake Turkana predates colonial penetration of the region, conflict between the indigenous groups and the Ethiopian government has regularly erupted since the beginning of the resettlement drive in the Lower Omo in 2005, both because the government failed to endorse strict boundaries to contain the encroachment of Konso farms on Bodi land, and because the new economic interests of the government in the region seem to have motivated a one-sided response to the conflict (Buffavand 2017). For example, in Bench Maji zone in 2012, government forces reportedly killed approximately fifty Suri people, following the deaths of three Dizi policemen marking land for the villagization of people who lost land to a palm-tree development (Hurd 2013). Adding to these tensions, local people have attacked townspeople and migrant workers near the new sugar estates, mostly in retaliation for the killing of their own people by fast-driving vehicles on the newly expanded road network (Buffavand 2017).

Certain conflicts are being exacerbated by the ecological changes underway in the borderlands between the Ethiopian and Kenyan states. These conflicts have historically taken the form of raids and attacks in the delta and lake margins (Lamphear 1992; Sagawa 2010). Elimination of the Omo flood and the decline of the lake’s fish populations are projected to increase the intensity and frequency of conflicts (Carr 2012).

In summary, the Omo-Turkana Basin is undergoing rapid and extensive change, with significant negative implications for people dependent on agro-pastoralism and fishing. These changes are occurring, however, alongside potential generation of new wealth. In the following sections, we consider how these impacts are likely to be felt across the different actors in the region. This involves recapitulating the major drivers of change, identifying potential thresholds, and naming current and future ‘winners and losers.’

## Discussion

### Ecosystem services

To synthesize the social and ecological impacts outlined above across such a large spatial scale, we employ an ecosystem services (ESS) model (TEEB 2010). This allows us to plot the changes in ecosystem services within a systems diagram, to identify which groups within the SES may be affected and to assess whether the changes are likely to be positive or negative in character. Framing the results through an ESS lens has the advantage of reducing the number of issues to be analyzed for different groups of actors within the region, in order to investigate variation in impacts across all groups. The model acknowledges three major categories of ecosystem services: regulating, provisioning, and cultural services (following TEEB (2010), we assume supporting ecosystem services are included within these categories as they underlie their production).

Regulating ecosystem services are those that ecosystems provide by acting as regulators, e.g., regulating the quality of air and water or by providing flood and disease control (TEEB 2010). Through the construction of the Gibe III dam, the mediation of the hydrological cycle within the Basin is altered from its pre-dam state, permanently dampening the hydrological cycle, i.e., reducing peak flows and flooding and increasing control of both water quality and quantity during floods and droughts. While this supports other ecosystem services (see provisioning services below), the pre-dam ecosystem was adapted to this hydrological variability, which increases connection with the flood plain and productivity, and thus the regulation may negatively affect nutrient flows and nutrient availability, reducing soil fertility and the primary productivity of Lake Turkana. It is also expected that pollination services will be reduced as biodiversity is decreased, and that biological control services will have to adjust to new levels of pest and vector borne disease, as seen elsewhere in Ethiopia, particularly with malaria rates near newly constructed dam reservoirs (Lautze et al. 2007; van Zyl 2015; Moges et al. 2016). Once expansion of the sugar and cotton estates is complete, the SES will settle into a new regime of regulating ecosystem services. For example, regulation by the dam will reverse some of the impacts of catchment degradation which occurs naturally and tends to make rivers like the Omo more ‘flashy’—i.e., liable to rapid flooding (Butzer 1970; Hopson 1982).

Provisioning ecosystem services are those through which ecosystems provide material or energy outputs (TEEB 2010). Gibe III will regulate the availability of water, thereby supporting irrigation. Given climate projections for the region show rainfall becoming more variable, increased regulation may be critical in supporting provision of water in the future (Jury and Funk 2013). However, increased irrigation has implications for equitable access to that water, and communities in the region practicing traditional livelihoods may see their water-based provisioning ESS diminished, firstly for agriculture, given reduced access to the river and reduced water quantity in the Omo and Lake Turkana once irrigation begins, and secondly for drinking water, as ground water levels change (Avery 2013). The kinds of crops cultivated in the Basin will also change dramatically, from food crops for consumption by local people, such as sorghum, to commodity crops for both domestic use and export, such as sugarcane and cotton. Irrigation and efficiency of scale will likely increase the sum output of provisioning ESS from the region but decrease the provisioning ESSs related to staple foodstuffs. Decreased quality of riverine forest and disappearance of the bush cover will reduce the provision of wild foodstuffs and will reduce the availability of raw materials for fuel, fodder, and medicinal resources for indigenous populations. Similarly, changes in water availability and land access for dry season grazing will negatively influence the rearing of animals in the Lower Omo and around Lake Turkana, reducing the provisioning ESS related to animal products. Early results show that reductions in nutrient inflow are negatively influencing productivity in Lake Turkana and reducing fish yields. Therefore, while provisioning ESS will increase overall, with benefits at the national scale, there will be a reduction in the provisioning ESS that local food systems are dependent on. Hence, this is likely to result in an increase in food insecurity, with resulting mobility, and the potential for further environmental degradation.

Cultural ecosystem services are non-physical services that the ecosystem provides to humans (TEEB 2010). Remodeling the landscape of the Basin is likely to precipitate displacement of indigenous groups, constraining physical interactions with the land, and divorcing people from their cultural heritage, their sense of place and belonging (Buffavand 2016; Stevenson and Buffavand 2018). There is potential for integration of displaced populations into alternative livelihoods e.g., jobs in the new plantations. However, the stress of such change can affect both mental and physical health (Snodgrass et al. 2016), and these impacts are likely to be exacerbated by the conflicts that are already occurring and anticipated to increase.

**Fig. 5:**
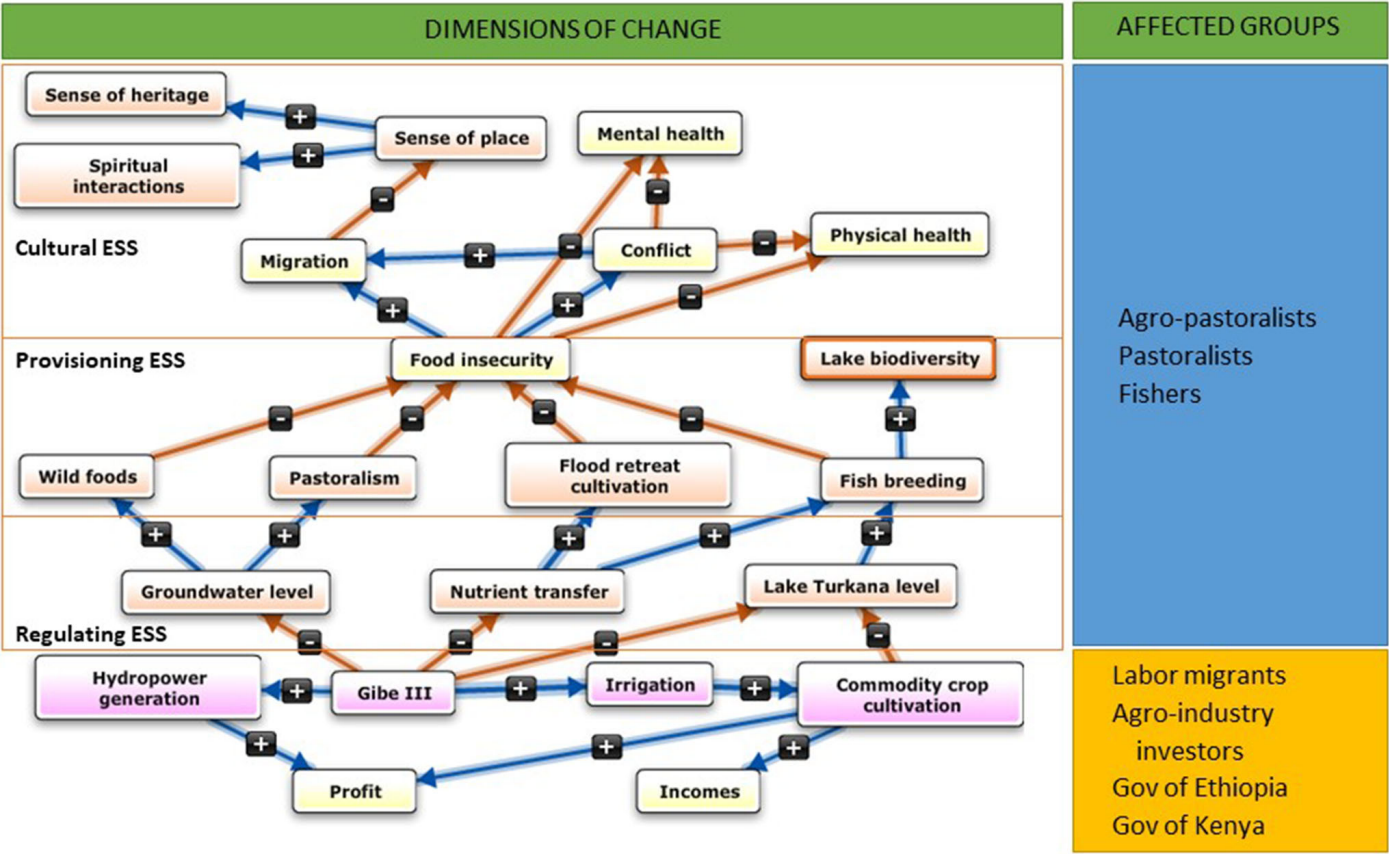
System diagram of the Turkana Basin, demonstrating technological developments (pink), system elements related to ecosystem services (orange), and key outcomes (yellow). Blue arrows indicate relationships are positive, orange arrows indicates relationships are negative

Figure 5 illustrates the relationships among key elements of the system and shows the cascading relationship between regulating, provisioning, and cultural ecosystem services. The following section builds on Fig. 5 to analyze the equity implications of changes in these ecosystem services.

### Equity implications

In analyzing the equity implications of social-ecological change in the Omo-Turkana basin, we have been guided by both O’Brien and Leichenko (2003) and Eames and Hunt (2013), who identify critical issues that influence equity outcomes for different actors, summarized as “the social, spatial, and temporal distribution of costs and benefits; access to and participation in decision-making and wider governance processes; who and what are afforded recognition in such processes; and whether wins and losses are voluntary or structural, relative, or absolute” (Hodbod et al. 2015).

We acknowledge that each actor group contains a diversity of perspectives and experiences. For the purposes of this study, we present results at a relatively low level of resolution as it is beyond the scope of this article to analyze fully the complex historical, political, and economic contexts of each actor group. Rather, we identify factors that shape the distribution of outcomes. While this risks losing the nuanced, contextual details inherent to, for example, a pure political ecology study, it strengthens a social-ecological systems approach by allowing conclusions to be drawn about the power imbalances of competing resource users who have different visions for what is desirable. The identification of winners and losers is based on our expert elicitation, as opposed to self-identification by the relevant actors. We consider a limited set of actor groups, namely (i) indigenous peoples (agro-pastoralists, pastoralists, and fishers); (ii) labor migrants; (iii) agro-industry investors; and (iv) the governments of Ethiopia and Kenya.

Thus far, the indigenous groups have borne the majority of costs in the development process, being impacted by changes in all three categories of ecosystem services. Agro-pastoralists in the Lower Omo have borne immediate costs, having experienced loss of traditional lands and flood-retreat agriculture with negative implications for both cultural and provisioning ecosystem services, resulting in a critical reduction in food security. Fishers are also bearing early costs, as lake levels recede and seasonal fluctuations decline, both of which reduce fish stocks through changes in the productivity of the system. Pastoralism is the most resilient livelihood option, being better adapted to drylands (Behnke and Kerven 2013; Hesse 2014) and thus far there is little evidence of pastoralists bearing costs. However, if plantations continue to expand, pastoralists are liable to suffer costs as access to grazing land and availability of groundwater diminishes: it is projected that the lack of seasonal inundation will mean groundwater stores are no longer replenished in the same manner, reducing biodiversity and availability of fodder species (Avery 2013; Carr 2017). Therefore, there is some differentiation of impacts depending on degree of dependence on different livelihood strategies. However, the majority of the region’s peoples depend on a diversity of livelihood strategies, rather than being specialists in either pastoralism or agriculture. For example, the Nyangatom depend on both herding and farming, and hence spread the risk of failure of either strategy in any given year. Permanently reduced access to flood-retreat farming or grazing land will therefore impact negatively on their resilience. There will also be further long-term costs to bear for all indigenous groups given anticipated changes in regulating ecosystem services. New roads should support access to markets and medical care but also increase risks of collisions involving humans and livestock. Lack of participation in decision-making places all indigenous groups at a disadvantage in the context of such rapid change.

Currently, labor migrants have received substantial benefits. Since the establishment of the sugarcane estates in the Lower Omo, the ESC has hired thousands of migrant laborers, mainly from Ethiopia’s southern highlands, and there is also a substantial Chinese presence associated with road and factory building (Kamski 2016a). While there is some conflict between migrants and indigenous populations, this has not affected the majority of laborers. The laborers are also dependent on ecosystem services for their food, water, and resources, but given their cash incomes, they can afford foodstuffs imported from outside the region.

Agro-industry investors are benefiting from the increased access to land, and there is a large potential for profit, but thus far, there is little evidence of large returns on investment (Kamski 2016a). This may be attributed to transport and communication infrastructure, which remains poor. In the future, the feasibility and profitability of commercial farming should increase; greater involvement in the decision-making process by this actor group is also likely to lead to a more favourable distribution of benefits.

The principal beneficiary of the ecosystem change is purportedly the Government of Ethiopia (GoE). Power generation from Gibe III began in September 2016, with 6 of the 10 turbines operational and generating at 800 MW (Wondimu 2016). There are profits to be made from sales of electricity from the Gibe III to the Government of Kenya and domestic customers, from land leases to private investors, and from the sales of sugarcane products. These benefits will therefore be passed onto both commercial and residential electricity consumers. However, the GoE may end up bearing significant costs. Both Gibe III and the Kuraz sugar development are behind schedule, and land suitability and the quality of soil are likely to be major limiting factors for irrigated cultivation of sugarcane and other cash crops (Kamski 2016a). In 2015, the ESC considerably down-scaled the KSDP to 100 000 ha serving four factories (as opposed to 175 000 ha and five factories originally envisaged) (see Kamski 2016b). Further, affected people in the Lower Omo require food aid, which must be delivered by either domestic or international agencies under the GoE’s remit.

The Government of Kenya (GoK) is thus far in an equivocal position. The commencement of power generation through Gibe III will potentially benefit Kenyans with grid access. However, ecological impacts on Lake Turkana also represent a substantial cost to the Kenyan economy. In recent years, less than 10% of the estimated potential of between 30 000 and 90 000 KT of annual fish production has been exploited (Avery 2010, p. 94, Avery 2013). The collapse of the fisheries would effectively rule out any future development of these resources and would require the GoK to provide food aid to increasingly large food insecure populations in the region. Exploitation of oil and wind resources in Turkana may offset these losses, but to date, a minority of Kenyans have monopolized the benefits of these developments. This is discussed further below.

To recapitulate, the clearest ‘winners’ at this stage are labor migrants, and the clearest ‘losers’ indigenous peoples (including both agro-pastoralists and pastoralists, but with more costs borne by agro-pastoralists currently). The other major actors—agro-industry investors, and the governments of Ethiopia and Kenya—are in an equivocal position, with potential for large gains, but also exposure to substantial risks. Important SES-wide impacts will result from changes in regulating, provisioning, and cultural ecosystems services, including potential environmental degradation; loss of biological and cultural diversity; heightened competition and conflict over natural resources; and the potential for increased dependence on food aid. There are, however, some ways of mitigating the costs of the Basin developments. In the next section, we explore these and appraise their likelihood of success.

### Options for mitigation of harmful impacts

A mitigation tool that would require a political shift within Ethiopia would be to prevent construction of further dams on the Omo (i.e., the Gibe IV and V), and reinvest resources in alternative development schemes. Potentially more feasible in the current political environment are smaller mitigation efforts. For example, in response to criticism of the initial environmental impact assessment commissioned for the Gibe III dam, the GoE proposed that the flood retreat cultivation would be sustained by simulated floods (Government of Ethiopia & Ethiopian Electric Power Corporation 2009). However, there is uncertainty over whether this will go ahead as described in the Environmental and Social Impact Assessment (CESI & Mid-Day International Consulting Engineers 2009) and there is no commitment beyond the short term. Releasing sufficient quantities of water from the reservoir would entail high economic costs (representing lost potential energy generation) and the irrigation infrastructure constructed at the headwaters of the Kuraz scheme might sustain damage (SOGREAH 2010). A lack of comparison cases (such simulated floods have rarely been attempted, probably because of trade-offs like those highlighted above) makes this a questionable solution to the problem of downstream impacts. The planned-release flood in 2016 was not judged by informants in the Lower Omo to be enough to sustain crops (L. Buffavand, personal communication, October 1, 2016).

There are a variety of mitigation options that could be implemented within the plantation schemes in the Lower Omo, including minimizing the irrigated areas and ensuring the most efficient irrigation technology and strategies are used. A farther-reaching change would be to repurpose irrigated areas to serve the interests of the local population. For example, if irrigation were used for production of fodder as opposed to cash crops, it could increase the productivity of the local livestock economy by reducing drought-time losses. Analysis of a parallel cases (including the transition from pastoralism to sugar and cotton economies in Ethiopia’s Awash valley) suggest that this might create a more sustainable increase in productivity than a transition to cash crop production (Behnke and Kerven 2013; Abbink 2014). Using irrigation for food crops would also balance out the productivity losses of communities previously reliant on flood-retreat agriculture, helping to increase food security. Neither of these measures would, however, address the problem of reduced flows to Lake Turkana.

Further technological developments in Kenya can be framed as potential solutions to livelihood challenges in Turkana, where ventures to exploit reserves of petroleum and establish large-scale wind farms are underway (Cormack and Kurewa 2018). The Lake Turkana Wind Power (LTWP) project, launched in 2006, aims to provide 310 MW energy to the national grid. In 2012, oil reserves estimated between 750 m and 1630 m barrels were discovered in the Lokichar region of southern Turkana (Ecofin 2016; Africa Oil Corporation 2017; Munda 2017; Cormack and Kurewa 2018). Both oil- and wind-power developments present challenges as well as potential benefits for local communities. Although the wind farm has created 699 local jobs and provision of carbon credit funds (projected at 2.3–6 million euros) (African Development Bank Group 2017; Lake Turkana Wind Power Ltd. 2017), it has also incurred loss of customary and legal rights to land and livelihoods (Sena 2015; Danwatch 2016). Discontent has manifested in regular organized action, and roadblocks outside oilrigs and workers’ camps and protests in various towns have become a common occurrence (Derbyshire 2016).

## Conclusions: challenges and opportunities going forward

The environmental and social impacts of current developments in the Turkana Basin are substantial. While provisioning ecosystem services and economic value can be created from large-scale cultivation, present designs will produce value at the expense of livelihoods for indigenous communities and the ecosystem services on which they rely. The analysis above shows that indigenous groups, who have been largely excluded from decision-making processes, will receive few of the benefits, but bear the majority of the costs, while migrant workers will be the main beneficiaries. Actors such as the governments of Ethiopia and Kenya stand to gain considerably from the hydroelectricity generated by the dam, but are also exposing themselves to significant risks, including the potential need to provide food aid or compensation for those whose livelihoods are affected. The Gibe III and plantation initiatives in Ethiopia present major challenges including the expropriation of land without compensation, and increasing difficulty in accessing vital resources, including water, forage, and wild foods. From a long-term, landscape-scale perspective, these deficits are only partially offset by new wage labor opportunities and resettlement schemes.

Given the scale of this development and the early phase of its activities, further research is required to assess social, spatial, and temporal trade-offs in order to identify more sustainable and equitable means of generating value. Important gaps in existing knowledge include (i) the productivity and value of large-scale cultivation and fisheries in this region, in comparison with traditional agriculture and pastoralism; and (ii) the volume and sustainability of groundwater resources within the Basin, on which the livelihoods of many people, especially pastoralists such as the Turkana, depend. Further, (iii) hydrological assessments are needed to investigate the cumulative effects of the existing and planned dams along the Omo (i.e., Gibe III, IV, and V). On the social and economic side, information is needed on (iv) the food security situation for people living downstream of the dam and those displaced by expanding estates; (v) the scale and character of migration activity in the region, especially among spontaneous labor migrants; (vi) changes in social identity due to changing livelihoods, cultural ecosystem services, mobility, and conflict; and (vii) epidemiological surveillance to assess and respond to changing patterns of disease associated with malnutrition and the newly introduced pathogens (e.g., HIV and other sexually transmitted infections).

Based on the development-forced displacement and resettlement (DFDR) literature, there are three further steps that could be taken to change the current trajectory to a more equitable one (Turton 2015). First, making all project assessments and feasibility studies publicly available would allow researchers to identify remaining knowledge gaps and to address the combined impacts of dam and irrigation development. Second, short-term provision of food aid would go some way toward mitigating the food insecurity situation that has been exacerbated by the developments. Finally, a targeted and well-funded program of compensation, livelihood reconstruction, and benefit sharing should be introduced. Such a program should focus on ways of integrating irrigated agriculture with subsistence farming and herding, with local communities taking an active, if not leading, role in identifying the most effective solutions strategies, and the government and NGOs playing a supportive and facilitating role, recognizing the adaptability and resilience of these communities in responding to environmental and climatic changes in the past. Only in this way are realistic pathways likely to be found that will result in sustainable and equitable solutions to current challenges. Given the pace at which change is occurring, such policies need to be developed and implemented as soon as possible.

## Electronic supplementary material

Below is the link to the electronic supplementary material.

Supplementary material 1 (pdf 231 kb)
