# Supplementary material for: Social-ecological change in the Omo-Turkana basin: A synthesis of current developments
Source: Ambio. 2019 Jan 8;48(10):1099–115. doi: 10.1007/s13280-018-1139-3 (PMC6722148; doi:10.1007/s13280-018-1139-3)
Supplement: Supplementary file 1 — Supplementary material 1 (pdf 231 kb) [file 13280_2018_1139_MOESM1_ESM.pdf]

Appendix S1 – Overview of data sources

Electronic Supplementary Material

This supplementary material has not been peer reviewed.

Title: Social-Ecological Change in the Omo-Turkana Basin: A Synthesis of Current Developments

Authors: Jennifer Hodbod*, Edward G. J. Stevenson*, Gregory Akall, Thomas Akuja, Ikal Angelei, Elias Alemu Bedasso, Lucie Buffavand, Samuel Derbyshire, Immo Eulenberger, Natasha Gownaris, Benedikt Kamski, Abdikadir Kurewa, Michael Lokuruka, Mercy Fekadu Mulugeta, Doris Okenwa, Cory Rodgers, Emma Tebbs.

Author	Discipline of study within the region	Data details	Related publications
Jennifer Hodbod	Environmental Social Science	Framing knowledge, social-ecological systems and political ecology work elsewhere in Ethiopia since 2011, and in the Lower Omo from 2018.	Hodbod, J., Tomei, J., & Blaber-Wegg, T. (2015). A Comparative Analysis of the Equity Outcomes in Three Sugarcane-Ethanol Systems. *The Journal of Environment & Development*, *24*(2), 211–236. https://doi.org/10.1177/1070496515583556
Edward Stevenson	Anthropology	Food security survey and ethnographic work in Lower Omo carried out between 2012 and 2018.	Stevenson, E. G. J., & Buffavand, L. (2018). "Do our bodies know their ways?" Villagization, food insecurity, and ill-being in Ethiopia's Lower Omo Valley. *African Studies Review*, *61*(1), 109-133.<br><br>Stevenson, E. G. (2018). Plantation Development in the Turkana Basin: The Making of a New Desert? *Land*, *7*(1), 16.
Gregory Akall	Geography (Human Development)	Fieldwork on the history of irrigation development in the Turkwel River Basin, Turkana County conducted between 2013 and 2016. Data methods involved participant observation, semi-structured interviews, participatory GIS, FGDs and documents analysis.	PhD thesis in preparation.
Thomas Akuja	Dryland Agriculture		Ebei, P.A., Gufu, O. and Akuja, T.E. (2008). Re-evaluation of long-term trends of drought early warning pastoral production indicators in Turkana District, Kenya 1987-2004, 2008, Droughts: Effects, Causes and Predictions (Ed. J. M. Sanchez), Chapter

			3:103-138.<br><br>Akuja, T.E., Mutua, B.M., Guliye, A.Y., Gichaba, C.M. Ngigi, M.W. Karachi, M. and Abdulrazak, S.A. (2009). Trends and Current Status of Indigenous Knowledge among the Inhabitants of the Turkwel Riverine, Turkana District, Kenya, Indilinga (African Journal of Indigenous Knowledge System), Vol. 8(2):209-217
Elias Alemu Bedasso	Social Anthropology	Historical ethnography, ethno-genesis, border narratives	Elias, A.B. (2015). “Surviving on the Margin: The Predicaments of Nyangatom along the Borderlands of Ethiopia, Kenya, and South Sudan”. Paper presented at 57th Annual Western Social Science Association (WSSA) Conference, November 26-28 2015. Association for Borderlands Studies.
Lucie Buffavand	Social Anthropology	Participant observation and interviews in Bodi, Selamago woreda, during four months between 2012 and 2015.	Buffavand, L. (2016). “The land does not like them”: contesting dispossession in cosmological terms in Mela, south-west Ethiopia. Journal of Eastern African Studies, 10(3), 476–493. https://doi.org/10.1080/17531055.2016.1266194<br><br>Stevenson, E. G., & Buffavand, L. (2018). “Do Our Bodies Know Their Ways?” Villagization, Food Insecurity, and Ill-Being in Ethiopia’s Lower Omo Valley. *African Studies Review*, *61*(1), 109-133.
Samuel Derbyshire	Social Anthropology, History, Archaeology	14 months of ethnographic fieldwork in southern Turkana 2014-2015 and various shorter	Derbyshire, S. (2017). Trade, Development and Resilience: An Archaeology of Contemporary Livelihoods in Turkana, Northern Kenya. PhD thesis, University of

		research visits including interviews, surveys and group meetings either side (2013-2017).	Oxford.<br>Derbyshire, S. (2019) 'Trade, development and destitution: a material culture history of fishing on the western shore of Lake Turkana, northern Kenya'. African Studies 78(1).<br>Derbyshire, S., and Lowasa, L. (forthcoming) 'The ruins of Turkana: an archaeology of failed development in northern Kenya'. In N. Berre, N. Hoyum, P. Geissler and J, Lagae (eds.) Forms of Freedom: Legacies of African Modernism. Bristol: Intellect.
Immo Eulenberger	Social anthropology; History; Development studies	>35 months of fieldwork in the region (2007-2017) with >1500 conversations recorded; extensive secondary data collection.	Eulenberger, I., Kamski, B., & Longole, H. (2018). Pastoral civil societies: cooperative empowerment across boundaries in borderlands of Kenya, Uganda and Ethiopia; study of civil society in Eastern African border regions.<br>Eulenberger, I. (2013). *Pastoralists, Conflicts and Politics: South Sudan's Kenyan Frontier*. In: C. Vaughan, M. Schomerus & L. de Vries (eds) "The Borderlands of South Sudan", New York, etc.: Palgrave Macmillan, 67-88
Natasha Gownaris	Aquatic Ecology/Fisheries	Three months of fieldwork on Lake Turkana over three years (2011-2013), six year collaboration with Kenya Marine and Fisheries Research Institute (2010-2015), literature review.	Gownaris, N. J. (2015). *Understanding the Impacts of Changes in Water Inflow on the Fishes of Lake Turkana, Kenya* (PhD, State University of New York at Stony Brook).<br>Gownaris, N. J., Pikitch, E. K., Aller, J. Y., Kaufman, L. S., Kolding, J., Lwiza, K. M. M., … Rountos, K. J. (2016). Fisheries and water level fluctuations in the world's largest desert lake. Ecohydrology.

			https://doi.org/10.1002/eco.1769<br><br>Gownaris, N. J., Pikitch, E. K., Ojwang, W. O., Michener, R., & Kaufman, L. (2015). Predicting Species' Vulnerability in a Massively Perturbed System: The Fishes of Lake Turkana, Kenya. PLOS ONE, 10(5), e0127027. https://doi.org/10.1371/journal.pone.0127027
Benedikt Kamski	Political Sciences; Hydro-politics	In-depth study of KSDP and selected commercial farming schemes in Nyangatom and Hamer Weredas. Extensive primary and secondary data collection in Ethiopia (esp. South Omo) and Lake Turkana region since 2013.	Kamski, B. (2016) The Kuraz Sugar Development Project *OTuRN Briefing Note Nr. 1* Omo-Turkana Research Network, Lansing Michigan. Retrieved from: http://www.canr.msu.edu/oturn/briefing_notes<br><br>Kamski, B. (2016). The Kuraz Sugar Development Project (KSDP) in Ethiopia: between "sweet visions" and mounting challenges. *Journal of Eastern African Studies*, *10*(3), 568–580. https://doi.org/10.1080/17531055.2016.1267602
Abdikadir Kurewa	Sociology; anthropology	Resident of Marsabit County and a research assistant at the National Museums of Kenya.	Cormack, Z., & Kurewa, A. (2018). The changing value of land in Northern Kenya: the case of Lake Turkana Wind Power. *Critical African Studies*, *10*(1), 89-107.
Michael Lokuruka	Food science, fish and food product quality assurance, history & food anthropology of Turkana region	Resident of Turkana County and worked in the past on the lake fisheries for over 8 years.	Lokuruka, MNI. (2015). a report to Turkana County Government on establishment of Lake Turkana Development and Management Authority, November, 2015.<br><br>Lokuruka, MNI. (2008). The failure of the Norwegian-supported fish factory in Turkana, Kenya: an ecological-historical perspective. Egerton J. Science and Technology, Vol 2&3, 147-165.
Mercy	Peace and Security	Political history of	Mulugeta, Mercy F. (2017) Small

Fekadu Mulugeta	Studies	Ethiopia, traditional governance systems, conflict in the Lower Omo and elsewhere in Ethiopia, government security providers in the Lower Omo.	Arms And Conflict Among East African Pastoralists: The Karamoja (In)Security Complex. *Africa*, 87(4), Oct 2017<br><br>Mercy, F.M. (2016). Statehood, Small Arms and Security Governance in Southwest Ethiopia: The Need for an African-Centred Perspective. In: S. Okello & M. Gebremichael (Eds.) “African-Centred Solutions: Building Peace and Security in Africa”, Addis Ababa: Institute of Peace and Security Studies, 103-134.<br><br>Mercy, F.M. Small Arms and Security governance: The Case of Nyangatom people of southwest Ethiopia, 2016. Unpublished PhD Dissertation<br><br>Mercy, F.M. (2015). The Future of Small Arms in Pastoral Lowlands of the Horn of Africa: a Case of Nyàngatom people in southwest Ethiopia. Peace, Federalism and Human Rights. Papers presented at the First Graduate Conference on Peace, Federalism and Human Rights, August 12-13, 2015. Institute of Peace and Security Studies (IPSS).
Doris Okenwa	Social & Cultural Anthropology, international development, natural resources, extractives industry	Ethnogaphy – 13 months fieldwork.	PhD thesis forthcoming
Cory Rodgers	Anthropology	18 months of fieldwork in pastoralist, fishing, and urban Turkana communities; 2 months of fieldwork at Kakuma refugee camps in Turkana County	PhD thesis forthcoming: The Politics of Inclusion and Negotiating Rightful Share in Kenya’s Emerging Oil Industry<br><br>Okenwa, D. (2017). Kenya's New Oil: Rethinking Brokers, Translators and Mediators (forthcoming

			AAS/ASA/ASAANZ 2017 conference)
Emma Tebbs	Remote sensing ecohydrologist	Satellite imagery from NASA's Landsat 8 and ESA's MERIS sensors.	Tebbs, E., Avery, S., & Odermatt, D. (2015). *Lake Turkana - Monitoring the "Jade Sea" from Space*. Retrieved from http://www.diversity2.info/products/documents/DEL8/D2Story-Turkana.pdf
